# Prohibition of Chloroform in Anæsthesia

**Published:** 1875-08

**Authors:** J. Hardman


### DENTAL REGISTER.

Vol. XXIX.] **AUGUST, 1875.** [No. 8.

### PROHIBITION OF CHLOROFORM IN ANÆSTHESIA.

BY J. HARDMAN.

*Read before the Iowa State Dental Society.*

One of the questions now engrossing the minds of thinking men in the medical and dental professions, is whether it be morally right to use chloroform for the purpose of inducing general anæsthesia.

In all the ages of the world where a sufficient degree of mental light prevailed to evince the presence of human reason, more or less effort has been made to palliate pain and suffering, and as man's advancement to a higher plane of intelligence has brought his physique to still an increased sensibility; so the efforts to combat pain and suffering arising therefrom, have in like measure increased as a consequence.

"Nature," it is said, "is her own interpreter." Hence we perceive she has for obvious purpose needed this increased sensibility to accompany as a handmaid of safety, to our very existence. But conceding that it be justifiable to mitigate pain by artificial means, how, as rational and responsible beings ought it to be done, and what means are we justified to use.

This opens up before us a field of serious thought, and one touching the interests of the dental practitioner, with much bearing, as he, in so many instances, has been a party to results most direful, and fruits to him, most bitter.

Man has ever sought to avoid pain, and it is reasonable that he will continue so to do. The excessive suffering attending surgical operations has excited inquiry as to some feasible means by which to avoid this dreaded consequence. From this cause was inspired the experimenting and final introduction of general anæsthesia. The first reliable agent of note that was introduced to the surgical world, was sulphuric ether, under the appellation of Letheon, about a quarter of a century ago. This agent did its work so well, and so exempt from fatal effects that the enthusiasm excited by the captivating eulogies of both surgeon and patient became the universal theme of praise, and at once the minds of many were turned to find something still more potent. The world had not long to wait, when chloroform was announced as the great anæsthetic catholican. It was pleasant, effective and claimed to be safe. To these two has been superadded another, namely, nitrous oxide. These three constitute the main and most reliable agents used.

It is not our present duty to examine each of these great agents of anæsthesia in detail, as there is now probably no one but that concedes that chloroform is infinitely more fatal than either the others. Had chloroform been introduced first, it is probable that from the bad effects attending its use, it would have very much lessened, if not altogether arrested the investigation further after such agents. Before chloroform was introduced, anæsthesia was rendered legitimate by common consent; there having been no discouraging results arising from the use of sulphuric ether, and an extensive use had already been made of it. No surgeon of note was found willing to operate unless aided by anæsthesia, and the same demand came from the patients. This, we say paved the way for the introduction and general use of chloroform; one amongst the most deadly agents admitted for the purpose of annihilating sensibility under the knife.

But few of us permit ourselves to reflect upon the modus operandi of general anæsthesia, we seldom associate it with death, yet, if the phenomena it exhibits are not those of death in extent, they certainly are in kind. Probably metaphysical philosophy will never clearly explain articulo mortis. We may as well now, as has been done heretofore, regard it as the final change—meaning the entire cessation of mental and physical consciousness. This final close of life is from the inbred faculty of vitality, one looked upon more or less with feelings of the deepest awe. If this principle of love for life and consciousness did not prevail, our very existence would be hazarded. Hence the justice in placing adequate penalties upon homicide and crimes tending to destroy human life.

We observe that this estimation, placed upon human life, is high or low in a people as the grade of their culture and moral intelligence is high or low. Hence the intensity of means used by a nation to preserve life marks indirectly its high degree of intelligence and moral worth. It follows then that we regard the placing of a low estimate upon human life, as a mark of a low state of intelligence, morals, and that a people practicing this nether strata of morals is a people subject to frequent murders, suicides and all evils that follow bad legislation and imperfect laws.

That there is no class of persons exerting as much influence in shaping sentiment upon this feature of morals in communities as do physicians, surgeons and dentists, we can readily admit. Therefore, how incumbent upon us to see that nothing in our practice may thus tend to influence aught but that which is truly allied to our high and beneficent calling.

That to take life wilfully is the worst type of crime; yet, nevertheless, to do so through wanton carelessness, or through inexcusable ignorance, exhibits a horrible infraction, and must command the severest of censure, and should not under our system of laws, go unpunished.

It is true, that among many of the usual avocations of life, fatal results often occur, such as collisions, etc., upon railways, explosions, etc., in steamboating, running away of fractious horses, etc., etc. And it may be asked, can the law interfere by prohibiting these modes of locomotion? We answer nay. These are only incidents and accidents in modes of travel, etc. where, in all ages, to accomplish the same unavoidable results, like fatal occurences have always more or less attended, and that the mode of locomotion, etc., exempt from these casualities has never as yet been discovered. Not so with anæsthesia by chloroform. Before its introduction we had ether, and more latterly is added nitrous oxide, means fully proven to be immensely more safe.

Suppose it was known, beyond a doubt, that a certain kind of steam boiler was subject to twenty explosions to one of other kinds in use, that do equally the same service in every other way; would it be rational and moral to still continue the use of the dangerous variety? And if still this fatal variety is manufactured, and parties persist in still using it to the iminent danger and sacrifice of the lives of innocent parties, would it not be right for the authority of the law to take cognizance of it, and enforce by adequate penalties a cessation of the further use of such a destructive machine; this is a parallel case; and it becomes the more forcibly so, when we remember that the law of our land does take cognizance of these things by requiring strict inspections, records adequate qualifications of engineers, etc., etc.

Whenever in practice the action of man extends to, and effects his fellow man, said action becomes amenable to law. Man is in this country a free agent to what is right. But to do *right* will never permit him to trifle with the life or welfare of his fellow man, even though his victim invites him to do so. Men have been implored to take the life of their fellow man, but not in any instance does the law contemplate its legitimacy. Infraction through ignorance of law may palliate the penalty but does not satisfy law.

Men may agree mutually to fight a duel, and although but few lose their lives in this way compared to the fatality from the use of chloroform, yet laws are enacted to punish duelling, and we approve this, and pray for the strict execution of the law.

The claim that the anæsthetic use of chloroform is beneficial and fully justifies the means to the end, we cannot for a moment entertain. Some force of logic would we admit, appear in this had we not means as adequate and infinitely more safe at our command. But this we certainly have in two or more well established agents.

Staticians differ so widely in reference to the number of fatal cases occurring annually from the effects of chloroform that we are constrained to the opinion that no correct record can be had, some putting it as low as ten, while others claim its having reached as high as one hundred and ten in the United States in a single year. The first is evidently too low and the latter no doubt too high as a criterion; but it is very evident that the public records do not by any means see near all the cases. Various causes tend to the avoidance of the reports, especially is it so in general surgery; many prefering to report the case as "dying under the operation," believing it less injurious to their reputation and they being less liable to a nauseous report from the coroner's jury.

We venture the assertion that all the cases of fatality from the use of sulphuric ether during the past twenty-five years has not reached one-tenth that resulting from the use of chloroform in a single average year.

At all events it is patent to every live member in our profession, that many are the victims to this agent. That none of us are in the possession of means to prevent disaster, if we use it. That no reliable antidote or resuscitant is offered, or can be anticipated. That the most healthy and well organized constitutions seem as liable to its deadly influence as the very frail. That no compounding with other drugs, or any peculiar mode of exhibition is a security. That when fatal results occur, the reputation of the administrator, the operator, the assistant and the profession to which they pertain, all are at the mercy of popular opinion, and more or less answerable to the courts of justice. That, indeed, from its known treachery, none are, who use it, exempt from unmeasurable blame.

These, with many more causes that might be adduced, force a conviction upon us, to the end that the only true and honorable way left for the profession in Iowa, and indeed, in the whole world, is to strictly refuse to further use this agent upon our patients for the induction of anæsthesia.

It is such questions as this, in our practice, that we, while in collective capacity, should well consider, and any errors of theory found amongst us at once set aright by a united expression of free sentiment thereupon.

Gentlemen, permit us then, to entreat of you, for the good there is in holding sacred that which is right; permit not this opportunity to pass without the placing of a united condemnation upon this dangerous practice, that will have no uncertain character.

